# Reviews

**Published:** 1864-02

**Authors:** 


### REVIEWS.

*Outlines of the Chief Camp Diseases of the United States Armies, as observed during the present War—A Practical Contribution to Military Medicine.* By Joseph Janvier Woodward, M. D., *Assistant Surgeon U. S. A.; Member of the Academy of Natural Sciences, Philadelphia; of the Pathological Society, Philadelphia, etc., etc.* Philadelphia: J. B. Lippincott & Co., 1863.

The first chapter is devoted to introductory remarks upon the classification of diseases, the meaning of terms, etc. The second chapter is devoted to a consideration of the "Conditions Determining the Character of Camp Diseases." Section 1st, upon "Malarial Influence." Section 2d, upon "Crowd Poisoning." Section 3d, upon "The Scorbutic Taint." He says: these "three wide-spread and powerful influences underlie and determine the character of by far the majority of camp diseases in America. They represent in fact the effect on man of three categories of conditions—climate, mode of life, and food. Intermittent fevers may be named as the typical result of the first of these influences; typhus and typhoid fevers of the second; scurvy of the third, And these three influences, variously combined, will appear continually as determining or modifying conditions, if not always as the causes of all the affections considered in this book."

Chapter iii Camp Fevers, Chapter iv Intermittent Fevers, Chapter v Jaundice, Chapter vi Camp Diarrhœa, Chapter vii Camp Measles, Chapter viii Catarrh, Chapter ix Pneumonia, Chapter x Pseudo-Rheumatic Affections; these constitute the headings under which has been furnished the profession a very valuable treatise upon the diseases of the army. The author describes the symptoms, pathological conditions, causes and treatment with as much minuteness as is compatible with the size of the volume, and we think with an accuracy and truthfulness which could not be surpassed. The book is evidently designed to be practically valuable to the army medical officers, and certainly cannot fail of its object if it is appreciated as it deserves.

There is no work devoted especially to a consideration of these topics which more deserves the attention of the profession; to the Army Surgeon the work is invaluable, while the physician in civil practice will obtain most valuable ideas as to the manner of preventing epidemics, and the causes which operate powerfully to produce the most fatal forms of diseases. The diseases which prevail in the army are new, or mixed, and the great mass of the medical men who treat them, are inexperienced in their care; on this account especially will this book be of value to the Army Medical Staff.

One chief attraction, as noticed in the volume before us, is the rational and common-sense view of medication in these different forms of disease. The hygienic conditions necessary for the relief of these diseases are plainly indicated. Sufficient room, healthy location, and proper food, constitute the staples of treatment in the diseases of the camp; the causes are to be removed and the disease will abate.

This volume contains about 400 pages, and is for sale in this city by Breed, Butler & Co.

---

*A Manual on Extracting Teeth—Founded on the Anatomy of the parts involved in the Operation; the kinds and proper construction of the instruments to be used; the accidents liable to occur from the operation, and the proper remedies to retrieve such accidents.* By ABRAHAM ROBERTSON, D. D. S., M. D., *author of Prize Essay on Extracting Teeth, etc.* Philadelphia: LINDSAY & BLAKISTON, 1863.

We have in this little book all that physicians care to know about the medical or surgical treatment of the teeth. The instruments most proper to use in extracting teeth are described and represented, as well as some which never should be used for such purpose. Physicians in the cities and larger towns have for the most part given up this branch of business into the hands of the dentists, yet it is well for all physicians to understand when teeth should be removed, and the use of instruments suitable for making such operations; their opinions are often asked, and they should be competent to give an enlightened answer. This book affords all desired information upon the important subject of the teeth.

